# Using a photovoice methodology to explore the impacts of living with COPD on quality of life

**DOI:** 10.1080/17482631.2025.2608197

**Published:** 2025-12-26

**Authors:** Samuel Grimwood, Amy Baraniak, Mark A. Faghy, Emma Sharpe

**Affiliations:** a Biomedical and Clinical Exercise Science Research Theme, University of Derby, Derby, UK; b School of Science, University of Derby, Derby, UK; c School of Sport, Exercise and Health Sciences, Loughborough University, Loughborough, UK

**Keywords:** Chronic obstructive pulmonary disease (COPD), quality of life (QoL), photovoice methodology, lived experience, thematic analysis

## Abstract

**Background:**

Chronic obstructive pulmonary disease (COPD) is a chronic condition with a debilitating symptom profile that significantly impacts quality of life (QoL). While its physiological burden is well-documented, innovative qualitative approaches can offer valuable insights into the lived experience, and its broader impact on wellbeing.

**Methods:**

Eight participants contributed 67 photographs using a photovoice methodology. Participants were invited via social media platforms and patient support networks (Breathe Easy Networks). Each participant provided up to ten photographs illustrating the impact of COPD and completed a follow-up interview for contextual understanding.

**Results:**

Thematic analysis of interviews and images identified three key themes: (1) self-criticism, shame and emotional responses to COPD, (2) interactions and relationships with others and (3) strategies and methods to help with well-being and managing the impact of COPD. Breathlessness emerged as a pervasive experience underpinning emotional responses, shaping perceptions of loss, change and experience of others.

**Conclusion:**

COPD's impact extends beyond physical symptoms, encompassing psychological and social dimensions. Photovoice offered a novel lens to explore these complexities highlighting the importance of addressing self-criticism, shame, emotional responses and interactions with others in patient care. Interventions should consider both individual coping strategies and systemic factors influencing daily life.

## Introduction

Chronic Obstructive Pulmonary Disorder (COPD) is a chronic condition characterised by a wide-ranging and debilitating symptom profile that significantly impacts Quality of Life (QoL). COPD influences a variety of patient outcomes such as mobility, self-care, daily activities, feelings of pain and discomfort, psychological wellbeing (including anxiety and depression), and the ability to self-manage. As the disease progresses, COPD patients increasingly rely on care as they face more frequent exacerbations and hospitalisations (Borge et al., 2024). Activities of daily life such as household chores and climbing stairs become increasingly difficult, while breathlessness and feelings of fatigue are negatively impacted by poor-sleep quality (Vogelmeier et al., 2021).

Lazarus and Folkman’s Transactional Model of Stress and Coping (1984) provides a useful framework for understanding how individuals with COPD appraise and respond to the stressors associated with their condition. Here, it is suggested that stress is dependent on how the individual interprets the situation and their perceived ability to cope. In COPD, where symptoms are typically persistent and unpredictable, patients may experience heightened stress and emotional discomfort, particularly if they perceive their coping resources to be insufficient. This theoretical framework is supported by recent findings, which demonstrated in a large sample of psychosomatic inpatients that *perceptions of stress* had a stronger impact on their stress responses than the actual stressors themselves (Obbarius et al., 2021).

Social support also plays a vital role in improving psychological wellbeing and QoL in individuals with COPD. Evidence suggests that patients with stronger social networks are more likely to adhere to treatment, engage in self-care behaviours, and experience reduced disease severity (Lenferink et al, 2018). This research aligns with Bandura’s Social Cognitive Theory (1986), which emphasises the role of observational learning, social reinforcement and self-efficacy in behaviour change. Social support can therefore enhance self-efficacy by providing encouragement, modelling effective coping strategies, and reinforcing positive health behaviours. While these benefits are well-documented in relation to mental health and self-efficacy, the impact of social support on other health outcomes (i.e., overall quality of life, hospital readmission) remains less clear (Barton et al., 2015). Further research is needed to identify the factors that promote effective social support and to examine the characteristics and influence of social networks in the management of COPD.

To support QoL and daily functioning, a plethora of interventions (both pharmacological and non-pharmacological) are available, primarily aimed at reducing symptom prevalence and severity (Muralidharan et al., 2015). Typically, interventions are tailored to disease severity using clinical markers and patient-reported outcomes. Self-management strategies are also promoted to enhance coping and QoL, with social support and self-efficacy (the belief in one’s ability to perform specific behaviours) playing a key role. According to Bandura’s theory of self-efficacy (1977), individuals who believe in their ability to perform specific behaviours are more likely to engage and persist in those behaviours. In COPD, promoting self-efficacy through education, goal-setting and social support can therefore empower patients to manage their symptoms more effectively to maintain a higher QoL.

Despite these developments, the research exploring the lived experience of patients with COPD is limited. Understanding how individuals manage the condition and its impact on their physical, psychological and social wellbeing is therefore essential. Innovative qualitative methodologies have allowed researchers to generate methods to obtain a deeper insight and understanding of a patient's lived experience. Approaches such as photovoice bring to life and give a personalised ‘voice’ to patients' experiences (Burton et al., 2017), as they generate and interpret photographs to explain and illustrate their understanding of their experiences, values and beliefs (Beazley, 2008; Ruby, 1992). The use of images creates an opportunity for participants not only to identify and interpret the situations they encounter, but also to represent these experiences to others through photography (Wang & Burris, 1997). Each photograph carries meanings elicited by the individual participant, which are of particular interest (Plunkett et al., 2013). This process occurs independently of the researcher’s presuppositions (Brunsden & Goatcher, 2007). To date, there is one photovoice methodology study in the context of COPD. The findings showed that breathlessness had a significant impact on participants' everyday lives, requiring many changes and support for daily self-care tasks, with feelings of frustration, anxiety and isolation reported (Sumner et al., 2023).

The photovoice methodology has been used in other conditions to explore the daily experiences of living with multiple sclerosis (MS), specifically the *‘invisible’* symptoms that patients do not discuss with clinicians and healthcare professionals. The findings provided insight into the daily challenges of MS symptoms and the invisible symptoms that require support from clinicians. However, key findings related to the psychological impact that symptoms had on the patient’s quality of life, including their psychological well-being. This enabled a perspective that semi-structured interviews and questionnaires would not have provided. It is therefore important to incorporate visual methods within qualitative health research to amplify participant voices, with the potential to find new knowledge. Despite their importance, there is a lack of investigation in COPD and follow-up studies are needed to capture patients’ experiences as they happen, in order to understand the true impact that COPD has on quality of life (physically, psychologically, socially, economically) and the meaning of this on everyday experiences through a phenomenological lens (Manen, 1997). Accordingly, this study aims to utilise the photovoice methodology to explore the impact of COPD on quality of life in relation to symptom experience. This information can be used to increase the understanding of the impact of COPD on day-to-day life, to inform recommendations for holistic self-management strategies and to improve the quality of life for people living with COPD, which might apply to others with chronic conditions.

## Methods

### Participants

Following ethics approval by the College of Science and Engineering Research Ethics Committee at the University of Derby (approval number ETH2021-0357), eight participants with a confirmed COPD diagnosis were recruited for this study. A target sample size of eleven participants was established based on sample sizes commonly used in previous qualitative studies in this area (Genoe & Zimmer, 2017; Korn et al., 2017; Kouijzer et al., 2018). However, data saturation, defined as the point at which no new themes or insights were obtained reached after eight participants completed the process and recruitment was completed, in accordance with published guidelines for determining saturation (Fusch Ph D & Ness, 2015). Participants were recruited through previous involvement in research (*n* = 3) or via the British Lung Foundation Breathe Easy group (*n* = 5). All participants provided written informed consent via the online study platform Qualtrics (by selecting ‘agree’) after reviewing the participant information provided. Participant demographics are detailed in full in Table I. Briefly, here, participants were all White British, with the majority being female (*N* = 7) and aged between 60 and 77 years (M = 70; SD 5.52). Patients had a diagnosis of emphysema (*N* = 5) or chronic bronchitis (*N* = 2), and one patient did not know their diagnosis beyond having COPD more broadly. Most of the patients had a diagnosis of at least 5 years, and all but one had physical comorbidities (e.g., hypertension, heart failure, etc.). The majority had a history of smoking (*N* = 5), and four had experiences of pulmonary rehabilitation.

**Table I.** Detailed summary of participants.

Participant (pseudonym)	Age	COPD classification	Time since COPD diagnosis	UK location	Smoking status	Smoking years	Comorbidities	Current medication
Sue	71	Chronic bronchitis	10 years+	East Midlands	Ex-smoker	28.5	Heart Failure/Heart Disease, Asthma, Sleep Apnoea, Anaemia	Salbutamol, Tioropium, Steroid Theophylline, Antiobotics, Nebulised medicine (NM)
Jane	60	Emphysema	10 years+	Southeast	Never smoked		Hypertension, Asthma	Terbutaline, Steroid
Jacqueline	72	Don’t know	5**–**7 years	East Midlands	Ex-smoker	13	Hypertension, Asthma	Salbutamol, Tiotropium
Aimee	73	Emphysema	10 years+	Southwest	Ex-Smoker	43	Hypertension, Diabetes, Asthma, Osteoporosis, Anaemia	Salbutamol, Aclidinium, Steroid, Theophylline, Carbocisteine, Antibiotics, Ambulatory oxygen therapy (AOT)
Pauline	77	Emphysema	10 years+	London	Ex-smoker	40	Rheumatic disease, Rhinitis	Tiotropium, Steroid, AOT
Pat	65	Emphysema	5**–**7 years	Southeast	Ex-smoker	11.2	None reported	Salbutamol, Carbocisteine, Steroid, Antibiotics
Teresa	75	Chronic bronchitis	7**–**10 years	Northwest	Ex-smoker	36.2	Hypertension, Asthma, Osteoporosis, Stroke	Salbutamol, Tiotropium, Steroid, Theophylline
Philip	72	Emphysema	2**–**5 years	Northwest	Never smoked	N/A	Heart Failure/Heart Disease, Diabetes	Carbocisteine

### Study design and procedure

This qualitative study employed a photovoice methodology to investigate the impact of COPD on quality of life, adhering to the procedures outlined in (removed for anonymity), which served as the foundation for the current research. Photovoice involves participants taking photographs that represent experiences that are important to them and their condition, which are then discussed in a follow-up interview. Once an expression of interest was received, patient-facing documentation, including the participant information sheet, consent form, photovoice instruction handout and initial survey, was distributed via Qualtrics (Qualtrics, Washington, United States). Participants used their mobile phones to take photographs and share them with Signal (Mountain View, California, United States). Once received, all images were transferred to a personal Microsoft Word (Washington, United States) document that was also used as a handout for reference during the subsequent interviews. Images provided by participants were not subjected to a separate or formal visual analysis; instead, they were used to prompt discussion during the interviews, helping participants to articulate experiences and reflect on specific contexts. Following the completion of the photovoice period, participants were invited to take part in a sixty-minute interview to explore the context/meaning behind each photo. Although a formal interview guide was not employed, the interviews did follow a consistent structure: each photograph and its accompanying description were discussed in turn. The photographs were introduced during the interviews as elicitation stimuli, providing prompts that encouraged participants to expand on their experiences, clarify perspectives, and ground their reflections in concrete examples. As such, the photographs shaped the depth and direction of the verbal data that were ultimately transcribed and coded. Specifically, participants were asked to describe each photograph, its meaning, and how it reflects the impact of COPD on their quality of life. Participants were free to add details regarding COPD and their quality of life; often, the photographs and discussion reminded patients of additional factors they had previously forgotten when taking the photographs as part of the study. The absence of a predetermined interview schedule reflects the purpose and ethos of photovoice/photo elicitation, which prioritises participant autonomy (Pasco et al., 2024). This approach is intended to create space for individuals to articulate vulnerable, personal, and deeply meaningful narratives, thereby fostering a sense of empowerment. Interviews were hosted via Microsoft Teams (Washington, United States). Interviews were recorded via Open Broadcast Studio (Michigan, United States) and transcribed via Express Scribe (San Jose, California, United States).

### Analytic strategy

Reflexive thematic analysis (Clarke & Braun, 2017) was employed to identify, analyse, and interpret patterns of meaning within the data. The first author SG, who conducted all interviews, undertook an iterative process of familiarisation, inductive coding, and theme development, reflexively engaging with the data to generate meaningful interpretations. Photographs were used as discussion stimuli rather than as data in themselves (Latz & Mulvihill, 2017), enabling participants to articulate the phenomena and meaning of COPD and quality of life in their own voices. Themes were generated inductively, reviewed and refined to capture the essence of participants’ experiences, and interpreted through a phenomenological lens to explore both individual meanings and shared patterns (Van Manen, 2016). While frameworks such as identity theory, illness narratives, or stigma theory can offer valuable interpretive depth, this study did not adopt a theory-driven analytic approach. SG carried out the primary analysis, including coding, naming, and finalising themes, with AB assisting in refining and streamlining the final thematic structure due to the size of the dataset. NVivo software (version 15) supported systematic line-by-line coding and data organisation, facilitating iterative refinement of codes and the development of a coherent coding framework. Preliminary codes were exported to Microsoft Word for a more tactile, interpretative phase of analysis, during which related codes were clustered in spider-diagram format to explore relationships and emerging patterns. Provisional themes identified by SG were then critically reviewed with AB. This iterative movement between software-assisted coding, manual thematic mapping, and collaborative discussion enhanced reflexivity, credibility, and analytic rigour throughout the process.

### Reflexivity

The researchers acknowledge that personal experiences, professional background, and longstanding involvement with a local *Breathe Easy* support group shaped the analytical perspective of this study. Sustained engagement with individuals living with COPD, both prior to and during data collection, fostered trusting relationships that enhanced the depth and openness of participant disclosures. The first author SG further recognises that lived experience of depression, together with experience of clinical practice within the NHS, may have influenced interactions with participants and the interpretation of psychologically oriented narratives. Importantly, SG’s positionality as a clinician-researcher shaped the analytic lens: while clinical expertise provided sensitivity to patient experiences and facilitated rapport, it also carried the risk of interpreting narratives through a professional framework. This dual role required ongoing reflexive awareness to balance empathy with critical distance, ensuring that participants’ voices were prioritised over clinical assumptions.

To mitigate bias, SG employed open and non-leading questioning, maintained clear boundaries between personal experience and analytic decisions, and engaged in regular supervisory discussions to critically review interpretations. Reflexive consideration was also given to differences in rapport between participants previously known to the researcher and those without prior contact, acknowledging this as a factor shaping the richness of the data.

At the time of conducting interviews, SG held MSc and BSc degrees in Psychology. While younger than most participants (31 years old), SG’s White British background aligned with the majority of those who took part in the study. SG received training in qualitative methods when completing both Psychology degrees and applied reflexive thematic analysis in dissertation work at each level, establishing a strong foundation for the analytic approach adopted in this study.

## Results

**Figure 1.**
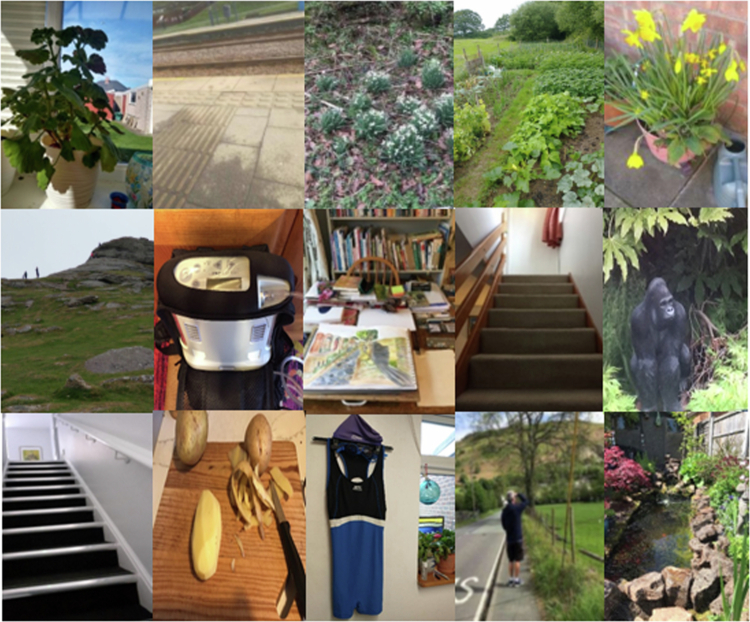
Summary collage of photos received from study participants.

**Figure 2.**
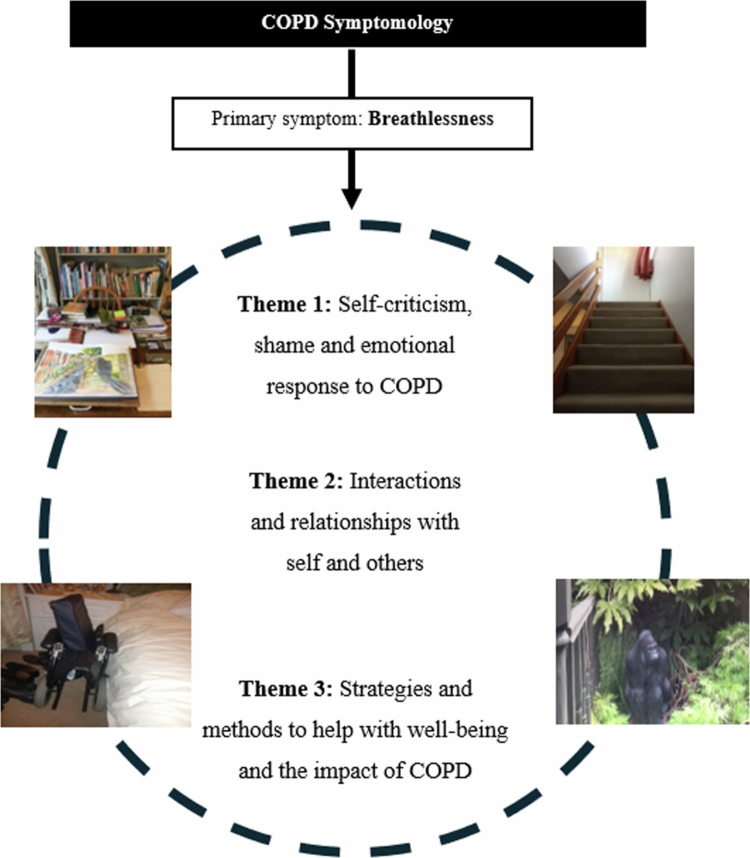
Thematic map showing themes and illustrative photographs.

Sixty-seven photographs (Summarised in Figure 1) were submitted and discussed across eight interviews. The focus of the interviews centred around a discussion of the photographs provided to explore the impact of COPD on quality of life, particularly in relation to symptom experience. This information was used to gain new insights into the coping strategies employed by COPD patients. Three themes were generated from the thematic analysis, including (1) self-criticism, shame and emotional responses to COPD, (2) interactions and relationships with others and (3) strategies and methods to help with well-being and managing the impact of COPD. Embedded throughout these themes is the experience of breathlessness, which underpins the emotional responses that patients feel in relation to loss, change and experience of others. Each of these themes is discussed, as evidenced by photographs and quotes from the patients. The themes are represented in a thematic map in Figure 2.

### Theme 1: self-criticism, shame and emotional responses

The primary emotion that was described by participants was the feeling of shame. It was pertinent that patients reported substantial changes in their behaviour to help them hide the impact their COPD had on them. This appeared to be an effort to avoid embarrassment, and the shame associated with the physical effects of COPD. Surprisingly, this included concealing the impact from work colleagues, family and strangers. People hid their condition through various behaviours, such as taking public transport instead of walking short distances (to avoid turning up at the office in a “dishevelled state”), stopping to look at and admire scenery to facilitate and hide necessary breaks from activities to reduce breathlessness, and pretending to cross the road to avoid being seen as “weird”.

Although evident across the data, Jacqueline provides explicit examples of how she actively plans strategies to hide her COPD from those around her (i.e., stopping to look at flowers or taking the bus) The purpose of this seems to be to maintain her “pre-COPD” identity, in addition to her dignity. However, even though she has implemented strategies to protect herself by reducing breathlessness (or the appearance of breathlessness) to manipulate what others think, she feels as though she is ‘cheating’. Jacqueline shared that prior to developing COPD, she would walk to work without relying on public transport or needing to avoid hills. Therefore, the feeling of cheating was in the sense of using a distraction to minimise the visible appearance to others of her COPD. Struggling to accept having a diagnosis of COPD, its associated disability, and the associated feelings of shame may result from a threat to the patient’s identity.

Stigma and isolation are commonly associated with conditions such as COPD and may play contribute to threats to personal identity. Individuals living with long-term conditions or disabilities are often perceived as ‘weak’ or fundamentally different from the rest of society, which can reinforce feelings of marginalisation (O’Donnell & Habenicht, 2022). This stigma adds to the existing shame and guilt associated with having caused the condition in the first place. Below, Pat discusses that COPD was the “new me” when diagnosed shortly after the loss of her husband. She initially felt defined by her condition, but through various circumstances, she experienced post-traumatic growth. She rejected the notion of being ‘a little old lady,’ which implied dependency and care. Instead, she regained her identity and social support, with her supportive networks understanding her needs without making her feel dependent on them.

*“At first, when you're first diagnosed, you feel you have this feeling, this is me, this COPD is me, but it's not you know I'm not my condition and yeah, and since then I now have a partner. When my husband died it was like I was a little old lady that had to be taken care of and go on holiday with things like that and I am far too young for that yeah so that the sort of two things are going on here all the time my husband dying and then me not long after being diagnosed yeah they are there if I should need them like my friends are but they are not sort of like worrying or anything important I think” Pat.*

**Figure 3.**
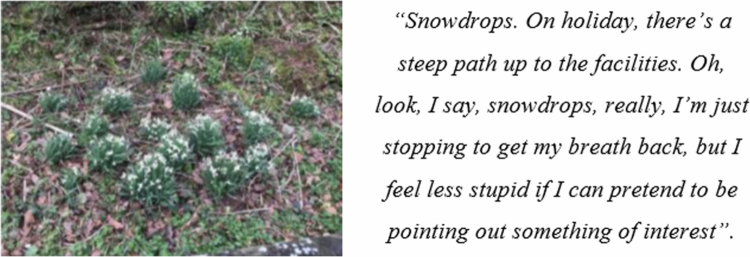
Photograph from Jacqueline of snowdrops and how she uses this to stop and catch her breath.

Jacqueline also describes clear examples of hiding her symptoms from her family. She describes how she hides her breathlessness from her family to avoid looking “stupid”. This is also depicted in one of her photos (Figure 3).

Patients conceal not only the physiological symptoms but also the psychological ones. They often avoid seeing friends when feeling low due to the impact of their COPD. Avoiding seeing others reduces social interactions and is a loss to the patient’s sense of belonging and connection. For Sue, it appears that only her husband sees the true extent of her COPD symptoms, which serves to preserve her dignity. This helps to maintain her identity and how others see her, preferring they remember her as she was before the COPD diagnosis and manifestation of her symptoms. For Pat, taking part in the Photovoice study brought the negative feelings about her COPD to the forefront of her consciousness, which had previously been ‘packed away somewhere’, hidden from both her and others. This helped to address these and recognise the impact COPD has on her psychologically.

*“Oh yeah, we have been married 50 odd years now… yes, he is the only one that sees me really down in the dumps, nobody else does, because if I don’t feel very good and want to see friends, I don’t go” Sue.*

### Theme 2: interactions and relationships with others

Several patients expressed that their breathlessness and reduced mobility resulted in friends and family treating them somewhat differently, compared to before their COPD diagnosis. Patients all said that friends and family were helpful and supportive. However, challenges emerged when the support made patients confront their illness identity, forcing them to acknowledge and accept their COPD and its associated limitations. Furthermore, patients expressed that there were times when they did not want assistance, support, or empathy from others, as it diminished their sense of independence and personal identity. However, they also acknowledged that it is difficult for their loved ones to see them struggling and their health deteriorating.

*“I'm really pleased that she's also a doctor, by the way but she doesn't comment. I hate people commenting I really hate people oh you ok you know do you want to stop for a bit (growls) (laughter) because that is part of me” Jacqueline.*

**Figure 4.**
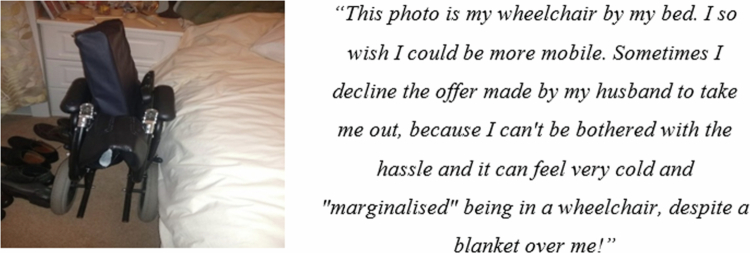
Photograph from Teresa of her wheelchair illustrating a lack of mobility and marginalisation.

Given the limitations caused by COPD, equipment such as wheelchairs and walking sticks are often needed to help patients engage in everyday activities and support their integration and inclusion in their social world (Figure 4). However, in some cases, these aids had the opposite effect. The need for aids can be difficult for patients to accept, exacerbated by anxieties about the potential perceptions of friends, family and members of the public towards them. For Teresa, the use of a wheelchair brought opportunities to increase mobility but resulted in frustration and anger. It often resulted in her being excluded from social interactions, as the wheelchair acted as a barrier to direct communication with her.

Jane has both congruent and incongruent views on how others behave towards her due to her COPD. On the one hand, and following the existing narrative, she sees that people want to protect her by offering support. However, Jane explains that this reaction stems from a fear of breathlessness, which is something the individual has not personally experienced, does not understand, and is afraid to be around. As such, Jane sees the support aimed at preventing breathlessness as somewhat selfish, with the primary motive being to avoid witnessing breathlessness rather than genuinely helping. Whilst previous participants reported actively hiding the breathlessness and coughing, Jane states that for her, COPD is an invisible and restrictive disability and not something that can be seen by others.

The role of close individuals providing social support for COPD patients is complex. This data reflects different types of support, such as protective buffering and overprotectiveness, which lead to poor outcomes; both types are associated with distress in patients with COPD, as they hide their symptoms and worry about their family’s response.

### Theme 3: strategies to help with well-being and managing COPD

All patients reported strategies to help them manage the impact of their COPD on their physical, psychological, and social well-being and quality of life. One of the most prominent discussions emerging from the data related to the challenge of managing emotional wellbeing. Given the emphasis placed on the emotional impact of living with COPD, this finding is perhaps unsurprising.

Implementing problem-focused strategies greatly assisted patients in functioning better, enhancing their quality of life and fostering a sense of independence. Key strategies included maintaining some physical activity, engaging in meaningful activities (e.g., gardening), utilising practical aids and accessing medical care and support, including pharmacology. Despite walking being difficult due to breathlessness and mobility issues, half of the group suggested that walking (if it is flat and has no steep inclines) helps to manage emotional well-being. This was especially important for patients whilst shielding during the COVID-19 pandemic. Some patients mentioned that walking gave them confidence that their COPD had not disabled every part of their daily life.

These activities brought a sense of purpose in the patient’s day-to-day life, which used minimal effort but had significant benefits.

*“I mostly go up get up get dressed and go into my little conservatory to have breakfast because I can see outside and it reminds me that I'm still part of the world and I can see every day the garden looks different and it is how I have made it in the last 12 years, there's always something to see and I find it lifts my mood takes me out of myself ” Teresa.*

**Figure 5.**
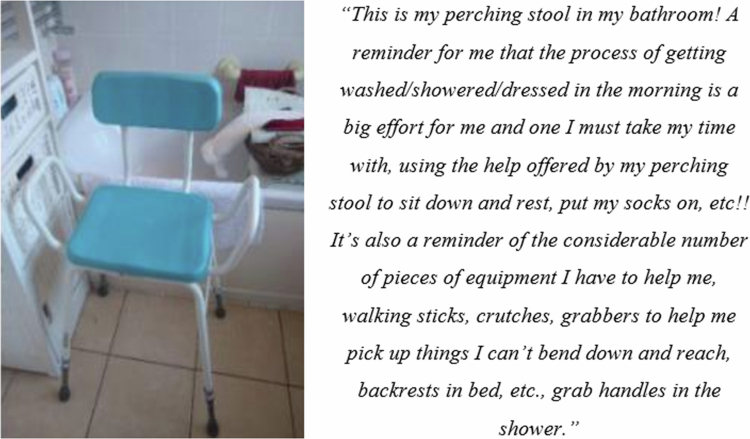
Photograph from Teresa of her perching stool which illustrates the challenges of completing everyday functional activities.

Teresa used her love of gardening to illustrate how she was impacted by COPD. She describes nurturing one of her plans over winter and the satisfaction she had that it didn’t die. During the interview there was a sense that the way Teresa treated her plants and garden reflected what she would like to experience in her own life, i.e., to feel connected with the world and be a part of it, to not feel isolated, to have a network of support, a change of environment and to be seen and to feel in the present moment again, despite the COPD symptoms. Whilst physical activity and being in nature with purpose was important, practical support interventions such as perching stools, shower handles, walking sticks, chair lifts, and wheelchairs were reflected in the photographs. Clearly, these physical aids contributed to a sense of independence for patients particularly in relation to mobility within and outside of the home (Figure 5). The aids reduced the amount of help patients needed to request and supported them to maintain their dignity, particularly relating to hygiene and bathing. These also provided a sense of reality for patients; the reminder that they must take time and use these things to enable them to do basic tasks.

## Discussion

This study aimed to explore the impact of COPD on the quality of daily life, focusing on symptom experience using photographs to capture a patient's lived experience through a contextual lens. Additionally, the study sought to uncover new insights into the strategies patients use to manage their condition daily. The resulting analysis derived three pertinent themes: *self-criticism, shame and emotional responses*, *interactions and relationships with others*, and *strategies to help with well-being and managing COPD*.

This study enhances a limited literature base that demonstrates a lack of self-compassion among patients with COPD. Specifically, the data indicates that a significant amount of self-criticism and shame stems from the belief that their condition is caused by their behaviours. Previous literature explores patients' experiences of shame associated with their smoking habits and the resulting impact on their daily lives, particularly due to COPD symptoms such as breathlessness (Halding et al., 2011). The current study builds on these findings by demonstrating that patients experience significant self-blame and stigmatisation for causing their COPD, along with profound shame due to the impact COPD has on themselves and their close family and friends (Jerpseth et al., 2021). Interestingly, Sigurgeirsdottir et al. (2020) observed that family members who care for people with COPD also report high levels of shame, especially where the patient seems unable to give up smoking. Future research should explore the role of shame linked to continued smoking-related behaviour and other COPD-related self-management behaviours. Feelings of self-blame, guilt, denial and shame also emerged from the impact of COPD in relation to threatening the patients’ identity, and patients being concerned about being perceived as ‘weak’. This is congruent with models of adjustment to chronic illness, such as Leventhal et al., (1992) and Moos & Schaefer, (1986). Hiding symptoms seems to be a part of living with COPD, with patients hiding their condition and its impact from anyone they feel might cast judgment (including friends, family and even strangers). This seems to help protect their identity and maintain their dignity. Patients often find breathlessness embarrassing and therefore try to conceal it. Hiding breathlessness and avoiding activities that trigger it due to feelings of shame and associated stigma is consistent with current literature (Breaden et al., 2019; Cooney et al., 2013). Pike (2018) described this behaviour within COPD patients as ‘shame-based avoidance’, which is often accompanied by feelings of guilt and embarrassment. There is a sense of anxiety when around others, which seems to be driven by feelings of self-criticism, self-judgment and shame.

Patients with COPD were also shown to experience changes in their interactions and relationships with others. Specifically, they reported shifts in how friends and family treated them due to their breathlessness and reduced mobility. While support from loved ones was generally perceived to be helpful, it was also shown to force patients to confront their illness, impacting their sense of independence and personal identity. Some felt that assistance could be overbearing at times, leading to frustration and a sense of exclusion, especially when using aids like wheelchairs. This study highlights the complexity of social support for COPD patients, demonstrating that overprotectiveness can lead to distress and a loss of independence, altering the dynamic between patients and their loved ones.

These findings align with Self-Determination Theory (Deci & Ryan, 1985), which emphasises the important of autonomy, competence and relatedness in psychological wellbeing. When support undermines autonomy (with individuals being overly controlling or intrusive), this is shown to negatively affect motivation and emotional wellbeing. Bandura’s concept of self-efficacy (1977) also comes into play here. Excessive assistance or support may signal to individuals with COPD that others may doubt their ability to manage, thereby reducing their confidence and perceived ability to cope with the condition.

These feelings of losing independence because of ‘over’ supportive family have been reported previously, resulting in a change in dynamics between COPD patients and close family and friends (Gabriel et al., 2014). The emotional burden on both patients and their caregivers is substantial, as caregivers may struggle with how best to provide support without being overbearing. This dynamic can lead to a strained relationship and increased stress for both parties. Future research should therefore aim to explore the experiences of carers and family members to gain a broader understanding of how COPD impacts not only the patient’s quality of life but also that of their wider social network. This approach can help to identify strategies to ensure family/caregiver support respects the patient’s independence while addressing the emotional needs of both patients and their caregivers.

The third theme focuses on strategies for self-managing COPD, and reveals various approaches, such as walking, practising mindfulness (e.g., watching fish in the garden), and using walking frames and handrails, that help individuals maintain dignity and manage daily life. These strategies are particularly valuable as individuals continue to experience significant levels of breathlessness and coughing, which worsen as COPD progresses. This leads to a loss of confidence, with patients needing support from loved ones and carers as they experience feelings of vulnerability and a diminished sense of self and identity. For example, while wheelchairs enable patients to remain mobile, they also cause them to be treated and perceived differently from others, which can be challenging. Maintaining independence is crucial for patients (Sharma et al., 2023). However, despite the variety of strategies employed, their effectiveness seems to diminish as symptoms progress. This leads to increased sedentary behaviour and a lack of support for psychological wellbeing during the later stages of COPD (Granados-Santiago et al., 2023). Further research, therefore, needs to focus on what strategies are useful for each specific stage of COPD, and how to support patients to best utilise the right strategies to enable patients to better manage their COPD for the best clinical and personal outcomes.

This study was the first to apply the photovoice methodology to a COPD population within the UK. Notably, only one other study has utilised this methodology in this context, conducted in Singapore, which focused on the impact of COPD on daily living activities (Sumner et al., 2023). In both studies, photovoice is shown to be an effective and highly beneficial research methodology, providing patients with a voice which allows them to contribute something different to the standard semi-structured interviewing, which, even with little structure, can be done in a way that imposes the researcher’s agenda. This methodology allows the agenda to be driven by the photographs provided by the participant, giving a different level of depth to the interviews than can be achieved otherwise. Photographs drawn from patients’ daily lives, which reflect aspects of their identity, can serve as valuable aids for those who struggle to verbalise or communicate (particularly in clinical settings such as interviews) by enabling them to express themselves more effectively. The novel contribution of this project lies in its detailed exploration of the lived experience of COPD, not only in terms of understanding symptoms but also in providing deeper insights into internally derived factors such as self-criticism and shame, which stem from key lifestyle choices.

There are limitations to using a photovoice methodology, including the challenge of capturing certain aspects of quality of life in photographs (e.g., the experience of fatigue). However, as demonstrated in this study, participants were able to provide detailed explanations for the photos that were shared, which adds additional context to the meaning of each image, enhancing the ability to share insight into the participants' thoughts and emotions. Furthermore, ethical constraints required patients to avoid taking identifiable images. This restriction, given the personal nature of their condition and the importance of support networks, made it challenging to capture meaningful photos. Future research may consider allowing patients to take personal and identifiable photos that, while not included in formal reporting, can support their commentary on how COPD affects them and the role of others in enhancing quality of life. The recruitment of participants through Asthma and Lung Foundation Breathe Easy groups may have resulted in self-selection bias, as individuals who engage with peer-support communities are likely to have distinct experiences, levels of disease acceptance, and willingness to discuss their condition compared to those less connected to such networks. A further limitation of this study is the small and relatively homogeneous sample, which restricts the extent to which the findings can be transferred to individuals with differing backgrounds, experiences, or healthcare contexts. The authors also acknowledge that their position as clinician-researchers with a vested interest in improving the quality of life for individuals with chronic conditions such as COPD informs their understanding and empathy towards participant experiences. Their professional backgrounds and personal experiences may have shaped the framing of research questions and the interpretation of data. However, to mitigate potential biases, the research team remained committed to authentically representing the voices of the participants by employing rigorous qualitative methodologies and engaging in reflexive practices throughout the research process. Given that this study was conducted in a single UK region with a relatively narrow demographic profile, the findings should be interpreted within this contextual frame. Variations in healthcare access, socioeconomic conditions, and cultural attitudes across different regions may shape COPD experiences differently, limiting the transferability of the results to wider populations. Finally, we acknowledge that phenomenological or sociological frameworks such as identity theory, illness narratives, or stigma theory could offer valuable interpretive depth, this study did not adopt a theory-driven analytic approach. As one component of a broader multi-study research project, the analysis prioritised generating clear, overarching themes to maintain coherence and consistency across studies, rather than engaging in the more intensive idiographic interpretation required by those theoretical traditions.

Future research should consider including carers or family members alongside patients with COPD to broaden the perspective on how COPD affects not only the patient’s quality of life but also that of family units/caregivers. For example, Reuss et al. (2005) conducted a study that interviewed family members to gather their perspectives on transitioning loved ones with chronic physical conditions into long-term care. Exploring a different perspective and narrative (i.e., beyond the patient) enables the experiences and insight which collectively help to increase the quality of care and support for the patient, as loved ones are often significantly involved in care. Reuss et al. (2005) highlighted the experiences of carers awaiting the transition from the family home to a formal care home. Their findings emphasised the decision-making process regarding care, the importance of communication, concerns about the support to be provided and the perceptions and attitudes of the patient during the move. Therefore, incorporating primary caregiver experiences should add further insight and detail into their realities of caring for a loved one. The data from both the patient and primary care caregiver could provide an in-depth bimodal perspective, with great potential for rich and detailed visual data, as well as follow-up interview discussions.

Overall, the findings of this study provide several recommendations for clinical practice and the psychological care of individuals living with COPD. Research has consistently shown that patients frequently experience illness-related stigma, often rooted in public and self-perceptions that COPD is a “self-inflicted” disease linked to smoking (Berger et al., 2011; Earnshaw & Quinn, 2012). Halding et al. (2010) found that many patients internalise these societal judgments, leading to feelings of guilt, shame, and diminished self-worth. Similarly, Harrison et al. (2017) reported that individuals with COPD often describe “feeling blamed” by healthcare professionals and the public, contributing to withdrawal, isolation, and reluctance to seek support. Such stigma can reinforce cycles of self-criticism and emotional distress, compounding the psychological burden of the illness and undermining engagement in rehabilitation and self-management programs (Cicutto et al., 2004).

Addressing these emotional and interpersonal dimensions is therefore essential for effective COPD care. Psychological interventions that target shame, self-blame, and self-criticism, particularly through compassion-based frameworks, may help patients manage internalised stigma and build resilience. A more compassionate, non-judgmental clinical ethos could counteract these stigmatising experiences and foster a sense of acceptance and trust. Acceptance and Commitment Therapy (ACT; Hayes et al., 2006) may be particularly valuable in helping patients acknowledge distressing emotions related to their illness while committing to behaviour changes aligned with personal values, thereby enhancing psychological flexibility. Compassion-Focused Therapy (CFT; Gilbert, 2009) similarly addresses self-criticism and promotes self-kindness, which can alleviate shame and improve emotional well-being. Integrating these therapeutic approaches into COPD care could therefore address not only psychological symptoms but also the broader impacts of stigma, ultimately improving patients’ quality of life and engagement with treatment.

## Conclusion

This study highlights the complexity of COPD, and whilst there are common themes generated from the experiences of various patients, everyone has unique needs that span physical, psychological and social dimensions. Findings reveal negative psychological outcomes linked to guilt from past health behaviours that may have contributed to the onset and progression of their lung condition. This guilt leads to behaviours which increasingly isolate patients from their social networks, thereby harming their social wellbeing. Adopting a novel approach to capturing lived experiences has enabled a deeper exploration of the impact of COPD on patients’ daily lives. By incorporating photographs, this method captures the nuances and realities of their lived experiences. It allows researchers and participants to extend discussions beyond the standard questions and conversations typically found in routine clinical practice. Addressing self-criticism, shame and various emotional responses, along with systemic factors such as interactions and relationships in the daily lives of COPD patients, is therefore crucial.

## Data Availability

Derived data supporting the findings of this study are available from the corresponding author [ES] on request. (a) Institutional Review Board Statement: The study was conducted in accordance with the Declaration of Helsinki and was approved by the College of Science and Engineering Research Ethics Committee at the University of Derby (approval number ETH2021-0357). See details under Methods [✓]. (b) The study received an exemption from an Institutional Review Board/Ethics committee; See details under Methods.
